# Oblique lateral interbody fusion combined with different internal fixations for the treatment of degenerative lumbar spine disease: a finite element analysis

**DOI:** 10.1186/s12891-022-05150-x

**Published:** 2022-03-04

**Authors:** Shuyi Zhang, Zhengpeng Liu, Chenshui Lu, Li Zhao, Chao Feng, Yahui Wang, Yilong Zhang

**Affiliations:** 1 Department of Spine Surgery, Affiliated Hospital of Chengde Medical College, Chengde, 067000 Hebei China; 2 School of Foreign Languages, Fuzhou University, Fuzhou, 350100 Fujiang China; 3 Department of Cardiovascular Surgery, Nanfang Hospital of Southern Medical University, Guangzhou, 510000 Guangdong China; 4 Department of Orthopedic, Chengde Central Hospital, Chengde, 067000 Hebei China

**Keywords:** Oblique Lateral Interbody Fusion, Finite Element Analysis, Internal Fixations, Degenerative Lumbar Spine Disease, Biomechanical

## Abstract

**Background:**

Little is known about the biomechanical performance of different internal fixations in oblique lumbar interbody fusion (OLIF). Here, finite element (FE) analysis was used to describe the biomechanics of various internal fixations and compare and explore the stability of each fixation.

**Methods:**

CT scans of a patient with lumbar degenerative disease were performed, and the l3-S1 model was constructed using relevant software. The other five FE models were constructed by simulating the model operation and adding different related implants, including (1) an intact model, (2) a stand-alone (SA) model with no instrument, (3) a unilateral pedicle screw model (UPS), (4) a unilateral pedicle screw contralateral translaminar facet screw model (UPS-CTFS), (5) a bilateral pedicle screw (BPS) model, and (6) a cortical bone trajectory screw model (CBT). Various motion loads were set by FE software to simulate lumbar vertebral activity. The software was also used to extract the range of motion (ROM) of the surgical segment, CAGE and fixation stress in the different models.

**Results:**

The SA group had the greatest ROM and CAGE stress. The ROM of the BPS and UPS-CTFS was not significantly different among motion loadings. Compared with the other three models, the BPS model had lower internal fixation stress among loading conditions, and the CBT screw internal fixation had the highest stress among loads.

**Conclusions:**

The BPS model provided the best biomechanical stability for OLIF. The SA model was relatively less stable. The UPS-CTFS group had reduced ROM in the fusion segments, but the stresses on the internal fixation and CAGE were relatively higher in the than in the BPS group; the CBT group had a lower flexion and extension ROM and higher rotation and lateral flexion ROM than the BPS group. The stability of the CBT group was poorer than that of the BPS and LPS-CTFS groups. The CAGE and internal fixation stress was greater in the CBT group.

## Introduction

In 1911, Albee [1] and Hibbs [2] described a posterior lumbar interlaminar fusion treatment for thoracolumbar tuberculosis. There has since been a trend towards using lumbar interbody fusion with similar surgical goals as the procedure described by Albee and Hibbs for cases of lumbar spinal instability. With the development of minimally invasive spinal fusion technology, several less invasive fusion methods, such as minimally invasive transforaminal lumbar interbody fusion (MIS-TLIF), extreme lateral interbody fusion (XLIF), and oblique lumbar interbody fusion (OLIF), have been developed in recent decades [3]. In 1997, Mayer [4] first described a minimally invasive anterior approach to the lumbar spine through retroperitoneal access at the L2-L5 level and transperitoneal access at the L5-S1 level. In 2012, Silvestre et al. [5] used a minimally invasive retroperitoneal anterior approach similar to Mayer's approach for anterior lumbar interbody fusion. This technique is referred to by Silvestre et al. as OLIF, which is an aorta-psoas approach. This novel fusion procedure was immediately recognized and adopted by spinal surgeons worldwide [6, 7].

There is a clear corridor on the patient’s left side from the L2 to the L5 vertebra including the skin, psoas and aorta that averages approximately 18 mm. This corridor can be further expanded to an average of 26 mm by blunt dissection of the muscle fibre and the gap between fascia [6]. This corridor allows direct access to the diseased disc without opening the spinal canal and damaging the posterior muscles, ligaments and bony structures; additionally, the OLIF technique substantially increases the support strength of the fusion because sufficient disc tissue can be removed, and the fusion has a large contact area with the endplate and can be extended to the sides over the dense bone protuberance surrounding the vertebral body [6, 8, 9]. Disruption of the psoas muscle and lumbosacral plexus can also be avoided by OLIF [10–12]. However, controversies regarding OLIF remain, such as whether internal fixation is necessary and what type of fixation is needed. Currently used surgical methods comprise SA and OLIF + UPS; OLIF + BPS or CBT are rarely reported, [6, 13, 14] and few researchers have investigated the biomechanical properties of OLIF with different fixation options.

FE analyses and cadaver experimental studies are complementary techniques for characterizing the complex biomechanical properties of the lumbar vertebrae [15, 16] Unlike cadaveric testing, the FE method can conveniently simulate different internal fixations and allows the determination of several values (including internal stresses and strains). Therefore, FE analysis has been used for decades in spinal surgery to find solutions for different problems. This study aimed to solve the problem of FE homology and clarify the suitability of new implants for clinical use via a finite element analysis (FEA) of the biomechanical differences between different device options for OLIF.

## Materials and methods

### Grouping

The following six FE models of the lumbar spine were created: (1) an intact model, (2) a stand-alone (SA) model with no instrument, (3) a unilateral pedicle screw model (UPS), (4) a unilateral pedicle screw contralateral translaminar facet screw model (UPS-CTFS), (5) a bilateral pedicle screw (BPS) model, and (6) a cortical bone trajectory screw (CBT) model.

### Construction of the intact model

A 38-year-old patient with degenerative lumbar spine disease was selected. A total of 481 computed tomography images (Siemens 128 slice 64-row, SOMATOM Definition AS spiral CT, Germany) with a slice thickness of 0.625 mm were provided by the Affiliated Hospital of Chengde Medical College. The computed tomography images were stored in Digital Imaging and Communications in Medicine format (DICOM). The collected raw DICOM data were imported into Mimics Research 21.0 (Materialise, Belgium) for three-dimensional (3D) reconstruction. Subsequently, the 3D model generated by Mimics was imported into Geomagic wrap 18 (reverse engineering software, USA). The noise and smoothing models were removed, and cancellous bone and posterior structure were created and imported into SolidWorks 2020 (CAD software, Dassault Systemes, USA). Articular cartilage and intervertebral discs were created, and the nucleus pulposus accounted for approximately 50% of the disc. The thickness of the cortical bone was 1 mm, and the thickness of the vertebral endplate and cartilage endplate was set as 0.5 [15–19]. Implant models (Table 1) were created simultaneously, and the lumbar spine model was assembled. (In this experiment, the lumbar spine of patients with abnormal lumbar intervertebral space height was not used, and the intervertebral space of the model was not modified. Since the intervertebral space is indirectly elevated to the ideal height or position by CAGE after OLIF, the model was directly used in such cases).

**Table 1:** Manufacturers and specifications of various different internal fixations

Implants	Manufacturers	Specifications (Unit: mm)	
Pedicle screws	Weigao,Shandong	L = 50	D = 4.5
		L = 45	D = 4.5
cortical bone screw	LIBEIER,Beijing	L = 50	W = 4.5
Facet screw	Weigao,Shandong	L = 44	W = 4.5
Cage	Johnson & Johnson (Ocrale)	L = 45	H = 9
		0°	
Rod	Weigao,Shandong	R = 5.5	

In specification, L, D, W, R represents Length, Diameter, Width, and Radius respectively

Before the implant was added to the patient's lumbar model, the nucleus pulposus of l4-5 in the model was completely resected by SolidWorks, and part of the annulus fibrosus and part of the cartilage end plate were removed. Allogeneic bone was implanted into the OLIF CAGE, which was then implanted into the surgically treated intervertebral space. This group was defined as the SA group. In the UPS group, pedicle screws and titanium rods were used to fix the left pedicle of the L4 and L5 vertebral bodies based on the SA model. The UPS-CTFS group was based on the UPS group, and the translaminar facet screw model was used from above the left lamina of L4 through the right inferior process of L4 to the superior process of L5 [20]. The BPS group received pedicle screws on the right side of the UPS model. The CBT group received bilateral cortical bone screws based on the SA group, and the CBT screws passed through three cortical bones [21].

### Grid division and boundary condition setting

These models were imported into ANSYS Workbench 2020 R2 (ANSYS, Ltd., Canonsburg, Pennsylvania, USA) for preprocessing, and corresponding material parameters were set for each component (Table 2) [16, 22–24]. Ligaments were simulated using springs subject only to pullout force (Table 3) [25–28].

**Table 2:** Material properties of each part of the FE

**Components/Materials**	elastic modulus **(MPA)**	Poisson ratio
cortical bone	12,000	0.3
cancellous bone	100	0.2
posterior structures	3500	0.25
anulus fibrosus	4.2	0.45
vertebral endplate	12,000	0.3
cartilage endplate	25	0.4
articular cartilage	50	0.3
titanium alloy	110,000	0.3
Cage (polyetheretherketone,peek)	3600	0.3
allogeneic bone	3500	0.3
nucleus pulposus	1	0.499

**Table 3:** FE parameters for each part of the ligament

Major ligaments	E (MPA)	A (MM^2^)	L (mm)	K = (A.E)/L (kg·M^−2^·S^−2^)
Anterior longitudinal ligament (ALL)	7.8	22.4	20	8.74
Supraspinal ligament (SSL)	8	10.5	22	3.82
Posterior longitudinal ligament (PLL)	10	7	12	5.83
Intertransverse ligament (ITL)	10	0.6	32	0.19
Capsular ligament (CL)	7.5	10.5	5	15.75
Interspinal ligaments (ISL)	10	14.1	13	10.85
ligamentum flavum (LF)	17	14.1	15	15.98

**Fig. 1:**
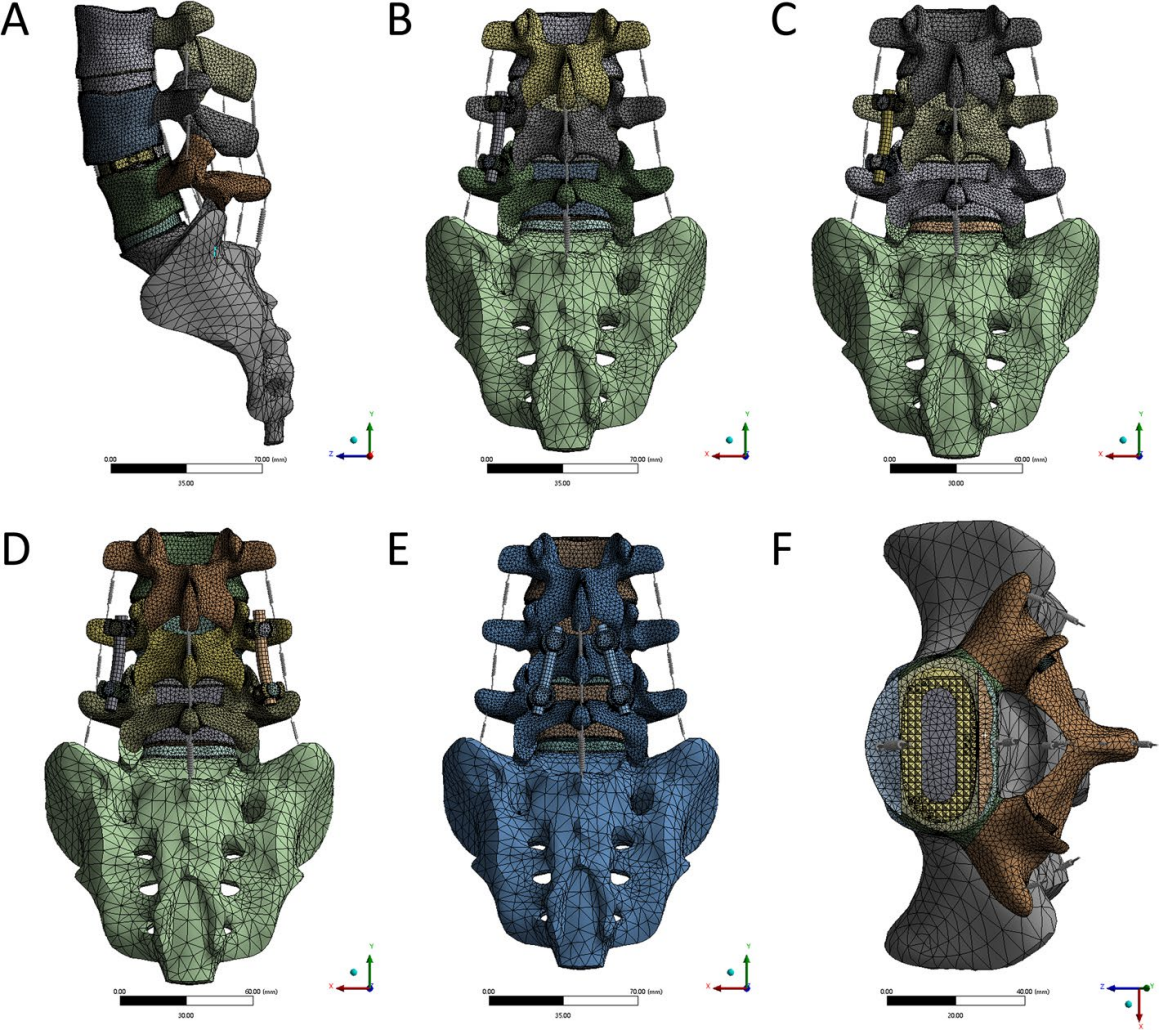
Model mesh division (**A**)SA model, (**B**)UPS, (**C**)UPS-CTFS, (**D**)BPS, (**E**)CBT, (**F**) The fusion cage in horizontal position

The number of nodes and elements in the model is shown in Table 4 and Fig. 1. The contact type of the facet joint was friction with a friction coefficient of 0.2. The remaining contact types were set to the binding mode. To improve the efficiency and accuracy of the calculation, the type was set to a tetrahedral grid, the size of the articular cartilage grid was 0.5 mm, the screw was 1 mm, and the rest of the objects were set to 2 mm.

**Table 4:** Number of nodes and elements after grid subdivision for various models

Model	Nod	Element
intact	498,843	304,822
SA	501,616	304,969
UPS	765,881	476,073
BPS	730,963	444,357
UPS-CTLFS	651,481	394,664
CBT	729,405	435,188

Six models were set up with boundary and loading conditions in the static model [16, 23]: bilateral alar sacralis fixation in S1, a vertical axial downwards preload of 150 N applied to the upper surface of L3 and a 10 N-m moment along the radial direction on the upper surface of L3 to simulate six different physiological motions. These motions were flexion, extension, right and left bending, and right and left axial rotation. The biomechanical stability of OLIF with different fixations was investigated by analysing and comparing ROM, CAGE and internal fixation stresses. The boundary condition settings for the intact model of flexion are shown in Fig. 1.

## Results

### Validation of the model

**Fig. 2:**
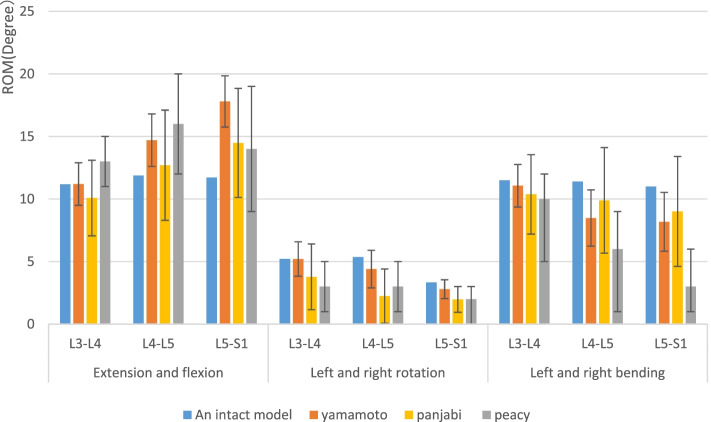
The comparison of the rom between the intact model and the previous in vitro experimental study. Note: The bars indicated standard deviation in experiment

After applying similar loads to our model, we compared our ROM results with those of a cadaveric study conducted by Previous in vitro experiments [29–32] et al. (Fig. 2). Our results were consistent with the previously reported data.

### Range of motion of the fusion segment [L4-5] (Fig. 3)

The ROM of the surgical model under a combined load of 150 N and 10 N-M is shown in Fig. 5. After insertion of the CAGE, the predicted ROM at surgical segment L4-L5 decreased in all motions in the intact model. Compared with all surgical models, the SA model had the greatest ROM in all modes of motion; the CBT model had the most restricted extension and flexion ROM. The BPS and UPS-CTFS model motions were similar, particularly rotation and lateral bending, and both sets of values were lower than in the other motion modes. In axial rotation, the CBT group was similar to the UPS group in terms of activity. In lateral bending, however, the CBT screws allowed more activity than UPS-CTFS and BPS but less than SA and UPS.

**Fig. 3:**
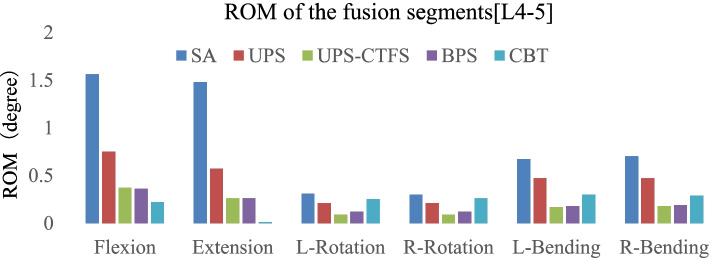
ROM of the fusion segments [L4-L5]

**Fig. 4:**
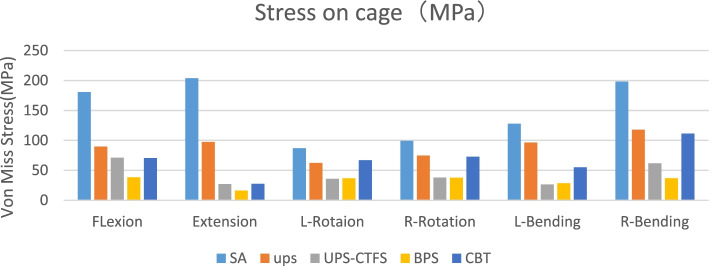
Stress on cage (MPa)

**Fig. 5:**
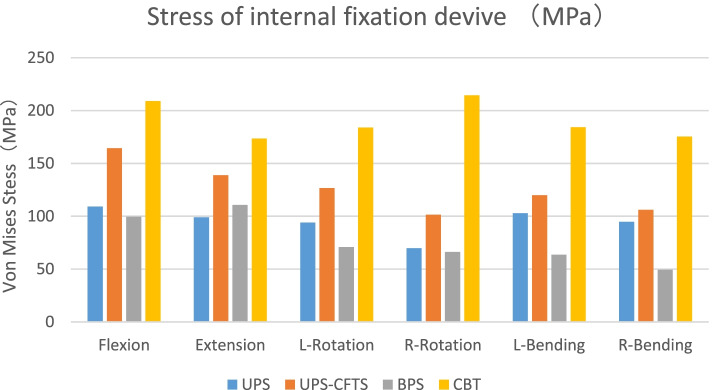
Stress of internal fixation device (MPa)

**Fig. 6:**
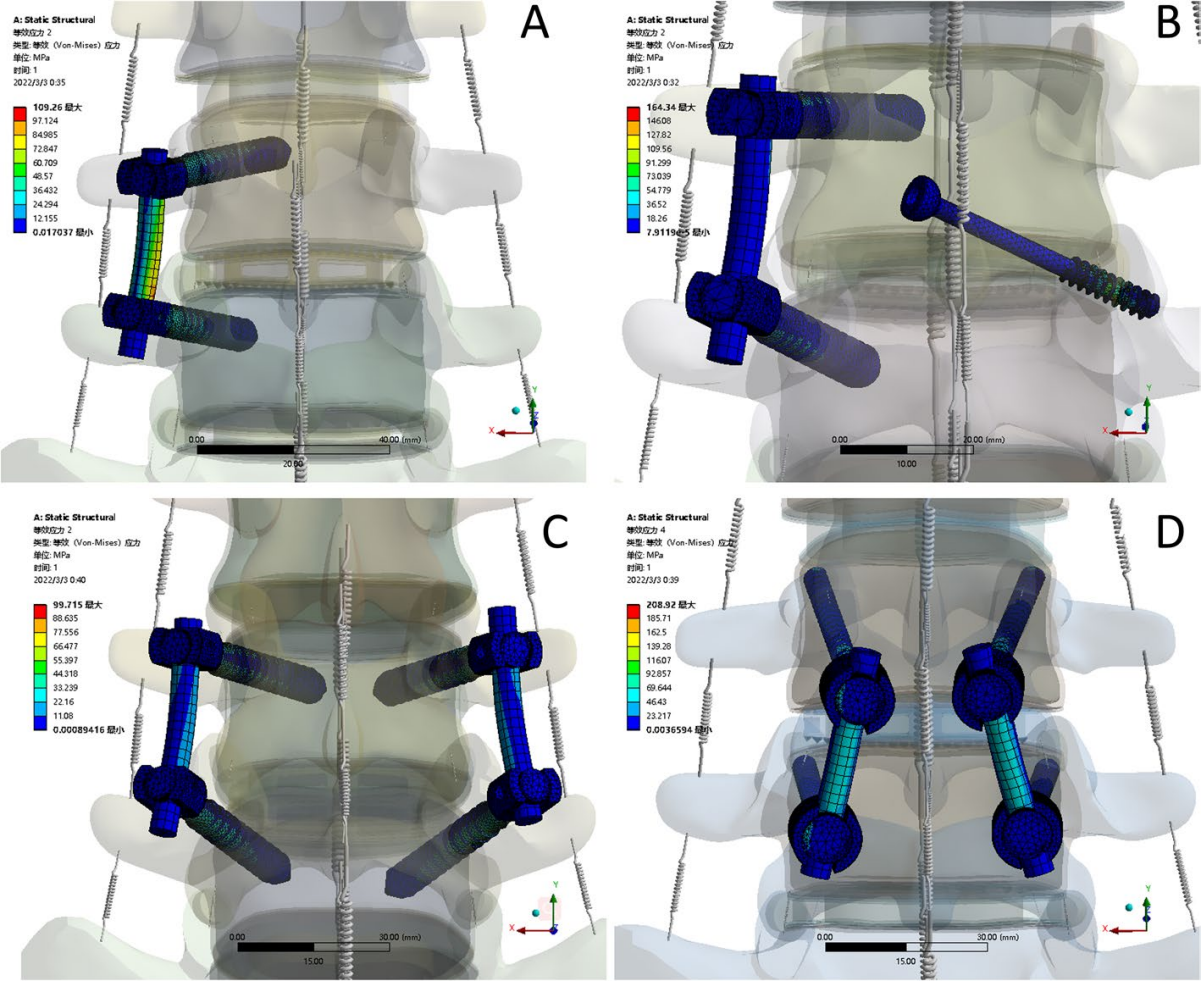
Von Mises stress of internal fixation during flexion in each group. **A** a unilateral pedicle screw model (UPS). **B** a unilateral pedicle screw contralateral translaminar facet screw model (UPS-CTLFS). **C** a bilateral pedicle screw model (BPS). **D** a cortical bone trajectory screw model (CBT)

### Von mises stress in CAGE (Fig. 4)

CAGE stress was highest in the SA model under different motion loading conditions. Compared with all the surgical models, the CAGE stress in the BPS model was the lowest without taking the left rotation into consideration. Considering left rotation, the UPS-CTFS CAGE resulted in the lowest level of von Mises stress, but the specific value was close to that of the BPS group. The CAGE stresses in the CBT group were greater than those in the UPS, UPS-CTFS and BPS groups in terms of rotation and left and right bending. In extension, the CBT group performed similarly to the BPS and UPS-CTFS groups. In bending, the CAGE stresses in the CBT group were greater than those in the BPS group but slightly less than those in the UPS-CTFS group.

### Von mises stress in internal fixation (Figs. 5 and 6)

The CBT internal fixation was subjected to the highest stress in all motions. UPS-CTFS was second only to CBT among all ranges of motion, and the BPS group had the lowest flexion, axial rotation and left and right bending internal fixation stress. In extension, UPS internal fixation was least stressed. Except for the UPS-CTFS group, the maximum forward-bending stress region of the other models was mostly located at the titanium rod. In contrast, the maximum stress in the UPS-CTFS group was located at the mobile area of the facet joint of the facet screw.

## Discussion

OLIF is increasingly being used by spine surgeons as methods and associated instruments are developed. In contrast to lateral lumbar interbody fusion (LLIF) and posterior lumbar interbody fusion (PLIF), OLIF accesses the target disc from the window between the major abdominal vessels and the psoas, thus reducing the risk for lumbar plexus injury and paravertebral tissue destruction [13]. OLIF is characterized by a shorter operation time, less bleeding, less postoperative pain, a shorter hospital stay and a faster postoperative recovery [33].

However, the difficulty of removing lateral recess, ligament flava hypertrophy, and hyperostosis of facet joints directly from the lateral corridor renders OLIF challenging. [34, 35] The improvement of symptoms after OLIF is based on the restoration of disc height, the increase of the foraminal area, the correction of coronal balance, and the indirect decompression of the neural elements by the wider implants [6, 36]. Joseph et al. [37] reported a complication rate of 20.2% (380/1885) after TLIF for nerve injury, while Abe et al. [13]  reported a complication rate of 1.2% (2/155) after OLIF, suggesting that the complication rate of OLIF for nerve injury is significantly lower.

Subsidence depends on multiple factors, such as the quality of the patients’ bones, the damage to endplates during their preparation, overdissection, multilevel fusion, small cages, and different types of instruments [38, 39]. Many spinal surgeons believe that different types of instruments are important factors for maintaining the stability of the surgical segment and reducing CAGE subsidence [3]. Although OLIF is an effective and increasingly performed treatment, there is still no consensus regarding the ideal instruments. Different fixation methods have been reported in the literature, including CAGE alone without other internal fixation devices, such as SA, UPS, and BPS. Relatively little has been reported on CBT with UPS-CTFS [36], and to the best of our knowledge, few studies have used FE analysis methods to analyse the biomechanical stability of OLIF with different fixation options.

Degenerative diseases of the lumbar spine, such as LSS, mainly occur at the L4-L5 segment, and OLIF is also commonly performed at the L2-L5 segment [6]. Due to the indirect decompression of the OLIF CAGE, we believe that an intact model simulation could be used to achieve the ideal vertebral space size. Therefore, in this study, the L4-L5 segment fusion model was used to analyse the effect of different implants after OLIF surgery. The OLIF CAGE can raise the height of the intervertebral space and expand the size of the intervertebral foramen, thus indirectly achieving decompression [40]. Therefore, we hypothesized that surgery could indirectly restore intervertebral height; We used patients with degenerative lumbar spine disease with relatively normal intervertebral height to implant OLIF CAGE and internal fixation devices to simulate postoperative patients with lumbar degenerative diseases.

### SA group

Selvon St. Clair et al. [41] performed an experimental biomechanical study of OLIF using cadaveric specimens and obtained dynamic and static biomechanical data. They found that the lumbar spine was comparable to the normal lumbar spine in terms of biomechanical performance standards after the OLIF procedure, while the ROM was decreased by more than 50%, indicating that the fusion segment was sufficiently stable to withstand a high degree of repetitive loading after the OLIF procedure. The studies of Shasti Mark et al. [40] showed that compared to the model with internal fixation, the SA model had the greatest ROM in all directions and the greatest CAGE stress, which may increase the potential risk for CAGE subsidence. Their studies also demonstrated that LLIF alone increases the stability of human spinal motion segments in all loading directions and that 30% of lumbar levels treated with LLIF show CAGE subsidence on imaging [42]. In addition, when subsidence of CAGE occurs, the possible clinical symptoms include axial pain and adverse neurological effects, potentially due to loss of indirect decompression space, collapse of the bone structure around the intervertebral body or degeneration. Tempel et al. [43] conducted a retrospective analysis of prospective data on 297 patients who underwent lateral fusion with SA. They found that fusion subsidence was a significant predictor of postoperative revision with the SA technique and recommended the implantation of internal fixation in patients at significant risk for fusion collapse. Most surgeons combine the use of internal fixation devices to maintain the stability of the surgical segment, reduce fusion loosening and promote intervertebral fusion [44]. Therefore, in many cases, such as in patients with osteoporosis [45], SA does not ensure stability and adjunctive fixation, such as pedicle screws, are required to distribute the load over the implanted vertebrae and avoid implant subsidence. In our experiments, the CAGE stress in the SA group was greater than that of the other groups with different motion loads, and the likelihood of postoperative CAGE subsidence was greater than with the other devices, but the relative ROM of the SA group was 25.58% of that of the normal model L4-L5 segment, indicating that SA reduces the relative ROM, limits the ROM of the fusion segment and provides a good fixation effect.

### BPS group and UPS

BPS fixation features a three-column concept of spine stability and is therefore the gold standard for the treatment of degenerative and traumatic spinal disorders; it is widely used as a posterior fixation device after OLIF CAGE implantation [46]. In our study, BPS showed the least ROM, CAGE, and screw stress in different motions, indicating that BPS provides the best biomechanical properties for OLIF. However, there are some disadvantages to BPS fixation, including damage to the paravertebral muscles during instrumentation and postoperative muscle atrophy, risk for nerve damage, vascular injury and increased operative time. In view of the relatively large size of the OLIF fusion and to reduce the risks associated with BPS during surgery, some surgeons choose UPS, which can provide better stability and reduce the cost of the procedure to the patient. UPS causes less damage to the paravertebral muscles, less perioperative bleeding and an overall lower implant cost. In our study, the relative ROM of the UPS group was higher than that of the SA group, but the ROM of the surgical segments was greater than that observed with the other internal fixation models. The stress on the CAGE in the UPS group was higher than that observed in the other groups, and there was an increased risk for CAGE subsidence. In a clinical trial, Aoki et al. [47] analyzed 1ss25 patients undergoing transforaminal lumbar interbody fusion and found that the incidence of CAGE loosening was higher in patients who received UPS (8.3%) than in those who received BPS (2.1%).

### The UPS-CTFS group

Translaminar facet screw fixation was developed by Magerl in 1984 and is used clinically for patients with acute spinal trauma and degenerative spinal disease [48]. Biomechanical studies have shown that the translaminar facet screw fixation technique provides a similar degree of spinal stability compared to conventional BPS [49]. Several studies have also demonstrated the biomechanical advantages of transforaminal pedicle screw fixation in terms of reducing flexion, extension and rotation [38, 50, 51]. Biomechanical and clinical studies have shown that UPS-CTFS can achieve stability and fusion rates similar to those of BPS [52–54]. In addition, the OLIF is long and wide and can be adjusted, and the CAGE can pass through the entire vertebral body and be fixed to the endplate bone surface with relative stability. We therefore speculate that relatively stable internal fixation can also be achieved with UPS-CTFS. In our study, we compared the BPS group with the UPS-CTFS group. The UPS-CTFS group had a more restricted extension, left and right rotation and left and right bending ROM than the BPS group; the flexion, extension and left and right bending CAGE stress in the UPS-CTFS group was greater than in the BPS group, but the two groups were similar in terms of rotation. The von Mises stress of the internal fixation device was higher in the UPS-CTFS group than in the BPS group, with better stress shielding. Therefore, we conclude that UPS-CTFS can achieve fixation results similar to those of pedicle screws in OLIF and can reduce the operative time. Thus, this method is worth applying clinically. In addition, BPS is more technically complex and invasive, requiring four pedicle screws and two titanium rods in a single-segment procedure, and causes damage to both lumbar muscles. This damage can be reduced when combined with percutaneous pedicle screw fixation. However, pedicle screws are more expensive than facet screws. Therefore, considering biomechanical stability, technical difficulty and cost, UPS-CTFS may be an beneficial alternative to the OLIF BPS and UPS procedures.

### CBT group

For the CBT technique, in 2009, Santoni [21] proposed a novel inferior-inferior to superior-inferior approach to fixation that increases the contact between the screw and the cortical bone. In contrast to the pedicle screw technique, this technique requires the screw to be inserted caudally to the cephalad in the sagittal plane and medially to laterally in the coronal plane, thus increasing cortical bone contact, maximizing tricortical fixation and increasing the holding screw strength. Santoni also stated that the CBT screw is more resistant to axial extraction but less resistant to bending and rotation than the BPS. CBT has a better effect in osteoporosis in terms of the resistance to pullout force and can better prevent the occurrence of internal fixation loosening. In our study, we found that compared to that of the BPS, the flexion and extension ROM of the fusion segment was relatively low with CBT screws, while in flexion and rotation, the ROM was greater than in the UPS-CTFS group. Additionally, the CAGE stress was greater in the CBT group than in the BPS group. Although the CBT group had the kinematic loads highest stresses among the models, these stresses were still below the yield strength of titanium of 897–1034 MPa [55]. Thus, due to their biomechanical properties, we conclude that CBT screws are a potential alternative to BPS, especially for patients with osteoporosis. However, the clinical use of this procedure still needs to be evaluated in a long-term retrospective study for comprehensive analysis.

### Limitations

Our study is based on FE analysis and has several limitations. First, the FE analysis was 3D modelled using CT data. We selected only the skeletal model; the remaining models constructed by later processing, and no tissue model, such as muscle or skin, was included, resulting in some experimental errors. Second, we only used the skeletal data of a single person for modelling and computing, ignoring differences among individuals. The description of provertebral disease was not considered in our study; thus, long-term clinical trials with large sample sizes and cadaveric experiments are required for validation.

## Conclusion

The BPS model provides the best biomechanical stability for OLIF, while the SA model is associated with a relatively high risk for postoperative collapse. UPS is an alternative internal fixation option due to its less invasive nature, lower cost, and ability to limit the movement of the fusion segment and reduce von Mises CAGE stress. Stresses were relatively high in the UPS-CTFS group compared to those in the BPS group, and there was a possibility of postoperative internal fixation loosening. UPS-CTFS and BPS result in a lower ROM of the surgical segments, leading to better stability. The unique path of the CBT screws results in better resistance to pullout force and limits the ROM of the surgical segment. However, in this experiment, the CBT group was subjected to greater stress than the other groups, and there may be risk for broken screws and rods in the postoperative period. Overall, CBT and the UPS-CTFS were found to be good alternatives to BPS, but a comprehensive and long-term clinical trial evaluation is required.

## Data Availability

The datasets used and/or analyzed during the current study are available from the corresponding author on reasonable request. Readers can access the data and material supporting the conclusions of the study by contacting Shuyi Zhang at 915,368,073@qq.com.

## Abbreviations

SA: A stand-alone model
UPS: A unilateral pedicle screw model
UPS-CTLFS: A unilateral pedicle screw contralateral translaminar facet screw model
BPS: A bilateral pedicle screw model
CBT: A cortical bone trajectory screw model
OLIF: Oblique lumbar interbody fusion
FEA: Finite Element Analysis
CT: Computed Tomography
DICOM: Digital Imaging and Communications in Medicine format
